# Genome‐wide association study identifies five new cadmium uptake loci in wheat

**DOI:** 10.1002/tpg2.20030

**Published:** 2020-06-24

**Authors:** Luqman Bin Safdar, Fakhrah Almas, Sidra Sarfraz, Muhammad Ejaz, Zeshan Ali, Zahid Mahmood, Li Yang, Muhammad Massub Tehseen, Muhammad Ikram, Shengyi Liu, Umar Masood Quraishi

**Affiliations:** ^1^ Department of Plant Sciences Quaid‐i‐Azam University Islamabad 45320 Pakistan; ^2^ Key Laboratory of Biology and Genetic Improvement of Oil Crops, Oil Crops Research Institute Chinese Academy of Agricultural Sciences, Ministry of Agriculture and Rural Affairs Wuhan 430062 China; ^3^ Plant Physiology Program, Crop Sciences Institute National Agricultural Research Centre Park Road Islamabad PO 45500 Pakistan; ^4^ Wheat Programme, Crop Sciences Institute National Agricultural Research Centre Park Road Islamabad PO 45500 Pakistan; ^5^ Department of Field Crops Ege University Izmir Turkey; ^6^ Statistical Genomics Lab College of Plant Science and Technology, Huazhong Agricultural University Wuhan 430070 China

## Abstract

Cadmium (Cd) toxicity is a serious threat to future food security and health safety. To identify genetic factors contributing to Cd uptake in wheat, we conducted a genome‐wide association study with genotyping from 90K SNP array. A spring wheat diversity panel was planted under normal conditions and Cd stress (50 mg Cd/kg soil). The impact of Cd stress on agronomic traits ranged from a reduction of 16% in plant height to 93% in grain iron content. Individual genotypes showed a considerable variation for Cd uptake and translocation subdividing the panel into three groups: (1) hyper‐accumulators (i.e. high Leaf__Cd_ and low Seed__Cd_), (2) hyper‐translocators (i.e. low Leaf__Cd_ and high Seed__Cd_), and (3) moderate lines (i.e. low Leaf__Cd_ and low Seed__Cd_). Two lines (SKD‐1 and TD‐1) maintained an optimum grain yield under Cd stress and were therefore considered as Cd resistant lines. Genome‐wide association identified 179 SNP‐trait associations for various traits including 16 for Cd uptake at a significance level of *P* < .001. However, only five SNPs were significant after applying multiple testing correction. These loci were associated with seed‐cadmium, grain‐iron, and grain‐zinc: *qSCd‐1A*, *qSCd‐1D*, *qZn‐2B1*, *qZn‐2B2*, and *qFe‐6D*. These five loci had not been identified in the previously reported studies for Cd uptake in wheat. These loci and the underlying genes should be further investigated using molecular biology techniques to identify Cd resistant genes in wheat.

**Abbreviations**
BY: biological yield
GpS: grains per spike
GWAS: genome‐wide association study
GY: grain yield
IWGSC: international wheat genome sequence consortium
LD: linkage disequilibrium
MTA: marker‐trait association
PH: plant height
QTL: quantitative trait loci
SL: spike length
SNP: single nucleotide polymorphisms
TGW: thousand grain weight
TN: tiller number.

## 1 INTRODUCTION

Cadmium (Cd) toxicity is detrimental to human health. Some of the adverse effects of Cd on human health are bone demineralization, kidney dysfunction, renal dysfunction, and an increased risk of lung cancer (Bernard, 2008). Polluted environment can be a direct source of Cd intake in humans which includes industrial exposure and intake through contaminated food. Industries (such as marble, steel, and aluminum) and the application of Cd containing phosphate fertilizers severely contaminate agricultural land and water resources (Grant, Clarke, Duguid, & Chaney, 2008; Singh & McLaughlin, 1999). The situation is more critical in the developing countries such as Pakistan, where the Cd concentration ranges from 0.02 to 184 mg/kg from normal soil to contaminated soil (Parveen & Shadab, 2012). Only in the last decade, the concentration of Cd in the polluted lands has increased by 200% in Pakistan (Waseem et al., 2014). As compared to other heavy metals, Cd has a better water solubility which enables it to percolate into the deeper soil layers thereby causing contamination of irreplaceable agricultural land and ground water resources (Nica, 2015). This high solubility of Cd also lets it easily absorbed by plant roots and rapidly translocated to the aerial parts via symplastic and apoplastic pathways. Consequently, it accumulates into the aerial parts of the plants including grains resulting in their contamination (Clemens, Aarts, Thomine, & Verbruggen, 2013). Nearly 27% of Cd intake through diet is attributed to grains and grain products (EFSA, 2012). Since wheat (*Triticum aestivum* L.) is the largest harvested crop worldwide, contamination of wheat grains by Cd would not only result in a food shortage crisis but can also prompt harmful effects on human health. Cd toxicity affects both morphological and physiological traits of plants such as biological yield, plant height, leaf area, number of grains, kernel weight, and grain yield. Oxidative stress, destruction of photosynthetic apparatus, genotoxicity, reduction in the root metabolism, and poor enzyme efficiency caused by high Cd have been known to cause plant cell death in various agricultural crops including wheat (Ci et al., 2010; Duan et al., 2010; Oncel, Keles, & Ustun, 2000), rice (Hassan, Wang, Ali, & Zhang, 2005; Lin et al., 2013) and maize (Andresen & Küpper, 2013; Schutzendubel et al., 2001; Xu et al., 2010).

**Core Ideas**

- Cd uptake shows a genotypic variation in the diversity panel, which subdivides the panel into hyper‐accumulators, hyper‐translocators, and moderate lines.
- Two Cd stress resistant lines (SKD‐1 and TD‐1) are identified in the wheat panel based on yield potential under stress.
- Five new loci for Cd, Zn, and Fe uptake in seeds are identified.

The uptake, accumulation, and translocation of Cd in plants are genetically controlled traits, hence vary among plant species and even between the various genotypes of the same plant species (Stolt, Asp, & Hultin, 2006; Verma, George, Singh, & Singh, 2007). Genetic approaches such as genome‐wide association study (GWAS) are often used to identify potential genomic regions controlling such variations so that the favorable alleles can be used to improve desired traits. These approaches have also been used for identifying Cd resistant genes in wheat but only a limited success stories are known. A major breakthrough in the research for Cd resistant wheat was achieved more than a decade ago when Knox et al. (2009) reported that marker assisted selection of single low Cd allele on chromosome 5B (*Cdu‐B1*) can significantly reduce Cd concentration in wheat plants. Recently, a Cd/Zn transporter *TdHMA3‐B1* has been cloned in the region of *Cdu‐B1*. The gene is reported to have two alleles: *TdHMA3‐B1a* and *TdHMA3‐B1b*. *TdHMA3‐B1a* is a resistant allele that transports Cd to the vacuoles in the roots, thus limiting its uptake and translocation to shoots. *TdHMA3‐B1b* has a 17‐bp duplication which makes it a non‐functional allele resulting in a 2‐ to 3‐fold increase in Cd uptake to the aerial plant parts in durum wheat (Maccaferri et al., 2019). Some studies have also identified genomic regions associated with Cd accumulation in bread wheat. Guttieri et al. (2015) identified five SNPs (IWA1439, IWA7579, IWA6681, IWA1752, and IWB43741) associated with grain Cd in multilocation trials on moderate‐Cd and low‐Cd locally adapted wheat varieties in Nebraska, USA. All these SNPs are in linkage with each other and map in the homologous region of *Cdu‐B1* on chromosome 5A in bread wheat. In another study, five marker‐trait associations (MTA) were identified for grain Cd on five different genomic regions including 1A, 2A, 2D, 3A, and 6D which do not colocalize with any of the previously identified loci (Bhatta et al., 2018). In a more recent study, Hussain, Campbell, Jarquin, Walia, and Morota (2019) have identified two loci for Cd on chromosome 2A and 2B by variance heterogeneity based GWAS, which determines the variability of trait rather than the mean value. These two loci also do not coincide with any of the reported loci. Such variation of the identified genomic regions in numerous studies attributes to the complexity of the trait in bread wheat. GWAS has also been effectively applied to identify genomic regions governing Cd accumulation and tolerance in other crops, such as *Aegilops tauschii* (Qin et al., 2015), *Oryza sativa* (Oono et al., 2014), and *Arabidopsis thaliana* (Chao et al., 2012). Here, we present a genome‐wide association of loci for Cd uptake in leaf and seed, Zn and Fe uptake in seeds, and grain yield related traits in a Pakistan historical spring wheat panel under Cd stress environment. Five MTA in our study (IWB29686, IWB39994, IWB2735, IWB12428, and IWB66179) colocalize with *Cdu‐B1*. However, the statistical significance of these MTA is relatively lower to pass the multiple testing correction. Five loci are identified as highly significant which are associated with Cd, Fe, and Zn uptake in seeds. These loci do not coincide with any of the previously reported QTL and should be further investigated by molecular biology techniques to identify causal genes for metal uptake and grain yield regulation under metal stress.

## 2 MATERIALS AND METHODS

### 2.1 Planting material and experimental design

The diversity panel used in this study consisted of 120 historical spring wheat lines adapted to irrigated and rain‐fed conditions of Pakistan (Supplemental Table S1). It comprised of 19 landraces, 30 green revolution lines (1965–1989), 32 post green revolution lines, (1990–2000) and 39 elite lines (2000–2013). The panel was planted for three years in field plots (2011–2014) at National Agricultural Research Centre (NARC) to check for phenotypic purity (Ain et al., 2015). The experiment was performed at the experimental field of the Department of Plant Sciences, Quaid‐i‐Azam University, Islamabad, Pakistan (33.7476° N, 73.1381° E). Special plots (4 m length by 4 m width by 2 m depth) with paved bottoms and sides were prepared for the field experiment. The soil for Cd stress plots was treated with 50 mg Cd kg ^−1^ soil, which is an average reported toxic concentration in the Cd affected soils of Pakistan (Waseem et al., 2014). The conditions of the Cd affected areas of Pakistan were replicated in the experiment. Three replications of 1 m row for normal (treatment 1 [T_1_]) and Cd stress (treatment 2 [T_2_]) conditions were planted in an augmented alpha‐lattice design. The dimensions of 4 m by 4 m plots were such that each plot had three beds of 1 m rows with each bed having 20 rows of plants. The row to row distance was 20 cm and the path between two beds was 0.5 m. Twelve plots were used for the experiment in total with each plot having 60 lines: 2 (plots/rep) × 3 (reps) × 2 (treatments) = 12 plots. Treatments and replicates were randomized within the plots. The bottoms and the sides of the plots were paved/cemented and a 2 m soil layer was added for planting. These precautions were taken to avoid the leaching of Cd into the deeper soil layers and to control the treatment conditions. Soil fertility level and texture were evaluated by analyzing three randomly collected samples (Chen & Ma, 2001). The tested soil had following characteristics: 3:3:5 ratio of sand, silt, and clay, 7.5 pH, 1:3 soil to water ratio, 0.8% organic matter, 3.3 dS m^−1^ mean EC value, 0.045 mg kg^−1^ phosphorus, 0.06 mg kg^−1^ potassium, and 0.73 mg kg^−1^ nitrogen. Seeds were surface sterilized with 4% H_2_O_2_ for 10 minutes and then thoroughly washed with double distilled water before planting. Thirty seeds from each accession were planted in 1 m rows.

### 2.2 Phenotyping

Phenotypic traits such as plant height (PH), grains per spike (GpS), tiller number (TN), thousand grain weight (TGW), grain yield (GY), spike length (SL), and biological yield (BY) were evaluated at the maturity stage following the methods of Pask, Pietragalla, Mullan, and Reynolds (2012). Chlorophyll index was measured non‐destructively at the mid position of the penultimate leaf using a portable chlorophyll‐meter (SPAD‐502 Minolta, Japan) at two growth stages (tillering [Chl__till_] and heading [Chl__head_]). Stress tolerance index (STI) was calculated for each cultivar following the method of Zanke et al. (2014):

$$\mathrm{STI}\;=\;\frac{\mathrm{GY}\;\mathrm{in}\;T_{1}\;\times \;\mathrm{GY}\;\mathrm{in}\;T_{2}}{\mathrm{mean}\;\mathrm{GY}\;\mathrm{in}\;T_{1}}$$

Here, GY for each line refers to the average GY of the three replicates of that line, whereas the mean GY in T_1_ refers to the average GY of all the lines in T_1_.

Reduction (%) of agronomic traits from T_1_ to T_2_ was marked as treatment 3 (T_3_) for the use in association analysis. Cadmium concentration was estimated from flag leaf (after anthesis) and seeds. The Cd concentration in T_1_ was either zero or negative for both leaf and seeds. Iron (Fe) and zinc (Zn) concentrations were also estimated from the seeds. Certified reference material (CRM) of duck weed BCR (BCR‐670; Sigma‐Aldrich) was used to verify the accuracy of the analytical equipment. The CRM and the plant samples (0.5 g dry weight) were digested by adding 7.5 ml of 65% HNO_3_ and 2.5 ml of 36% HCl (Merck) in CEM ‐ MARS 6 (microwave accelerated reaction system). Three digested samples from each replicate were analyzed using an atomic absorption spectrophotometer model WFX‐210 (Beijing Beifen‐Ruili Analytical Instrument Co. Ltd., China) to estimate the concentrations of Cd in leaf and Cd, Fe, and Zn in seeds. The atomic absorption results of Duck weed BCR (BCR‐670) CRM, that is, 20 ± 3 mg kg^−1^, 0.90 ± 0.01 mg kg^−1^, and 78.5 ± 2.1 µg kg^−1^ (Zn, Fe, and Cd, respectively) showed non‐significant differences with its certified values (24 ± 2.1 mg kg^−1^, 0.94 ± 0.04 mg kg^−1^, and 75.5 ± 0.5 µg kg^−1^ for Zn, Fe, and Cd, respectively), which suggested the accuracy of analytical instrument.

### 2.3 Statistical analysis

#### 2.3.1 Phenotyping

Descriptive statistics and a mixture‐design analysis of variance for alpha‐lattice design were performed in XLSTAT 2017 to evaluate the statistical variation between genotypes within a treatment and between two treatments (T_1_ and T_2_). Correlation, scatter plots, and histograms for all traits were visualized using GGally package in R.

#### 2.3.2 Genotyping and genome‐wide association analysis

Genotyping with Illumina iSelect 90K SNP array was reported in one of our earlier reports on the diversity panel (Ain et al., 2015). The SNP markers were aligned to IWGSC RefSeq v.1.0 to obtain their physical positions. Genotyped data had 81,587 markers which were trimmed for MAF < 5%, monomorphic markers, markers with missing data such as genomic positions, and markers with indels. Finally, 14,960 high quality polymorphic SNP markers were used for genome‐wide association study (GWAS). Population structure (Q) was estimated using STRUCTURE software while kinship matrix (K) was analyzed in TASSEL software. Linkage disequilibrium (LD) between marker pairs was estimated in TASSEL using the observed allele frequencies vs. the expected allele frequencies. LD estimation was based entirely on mapped markers. The critical *r^2^* value, beyond which LD is due to true physical linkage, was estimated by taking the 95th percentile of the square root transformed *r^2^* data of unlinked markers (Breseghello & Sorrells, 2006). The percentage of marker pairs significant at various *r^2^* values (0.2 and 0.2641) and *P* < .001 were estimated for all chromosomes to compare the degree of LD among genes. GWAS was performed using mixed linear model (MLM), the Q+K model, in TASSEL (Yu & Buckler, 2006). Significance of threshold for GWAS was set according to Bonferroni correction of α = 0.05 (0.05/14960) to account for false positives expected from the type‐I error during multiple testing.

#### 2.3.3 Identification of loci and candidate genes

Pairwise LD was used to estimate the linkage of MTA with the surrounding SNPs and to measure the distance of LD blocks. The LD block distance was considered as one locus. If the MTA had no linkage with any other SNP or if the block size was too small due to a small number of SNPs in 90K array, the LD decay distance around the MTA was considered as a locus to get the associated genes. Genes were retrieved using IWGSC RefSeq v.1.0 GFF3 version present in the EnsemblPlant database (http://www.ensemblgenomes.org/pub/plants/release-44/gff3/triticum_aestivum). Gene annotation was retrieved using EnsemblPlant and EMBL‐EBI (http://www.ebi.ac.uk/interpro) databases. Gene annotation was performed using BLAST2GO (Conesa & Götz, 2008) and was compared with orthologous gene functions from rice and arabidopsis for an accurate and reliable functional annotation.

## 3 RESULTS

### 3.1 Phenotypic evaluation and statistical analysis

Analysis of variance revealed genotypic difference in the panel within one treatment as well as between different treatments (Genotype × Treatment), suggesting a highly variable impact of Cd stress on different genotypes. Micronutrients and yield related traits were reduced significantly under Cd stress treatment (T_2_): Zn (−93.54%), Fe (−92.90%), GY (−68.22%), BY (−66.52%), TN (−51.06%), and PH (−16.01%) (Table 1). The genotypic variation for quantitative traits was extremely high, e.g., in case of PH, some genotypes showed a highly reduced PH: Shahkar‐95 (−45.4%), Tatara‐99 (−42%), Chenab‐00 (−36.6%), Rawal‐87 (−36.4%), and Chakwal‐97 (−36.4%); while some genotypes showed an increase: Seher‐06 (+13%), Kirman (+9.5%), Haider‐00 (+8.1%), Imad‐05 (+6.4%), D‐97 (+5.9%), Iqbal‐00 (+5%), C‐306 (+3.14%), Lu‐26 (+2.8%), Suleman‐96 (+2.7%), and As‐02 (+0.5%), under Cd stress. Cd stress showed a drastic impact on overall grain yield of plant in most of the genotypes e.g., DWR‐97 (−98%), Bars‐09 (−92%), Zardana (−91%), Lu‐26 (−91%), Barani‐70 (−91%), Sussui (−90%), and Chakwal‐86 (−90%). Only two genotypes in the panel showed resistance to Cd stress by maintaining their GY under stress, i.e., SKD‐1 (−2%) and TD‐1 (−5%). Some other examples of Cd impacts on plant yield were high percentage reductions in GpS and TGW. For instance, extreme reductions in GpS were observed in Drawar‐97 (−78.6%), Pirsabak‐91 (−73%), Zardana‐89 (−73%), and Chakwal‐97 (−70%) and TGW in B‐silver (−57%), Rawal‐87 (−36%), Kohinoor (−32%), Zarghoon‐79 (−31%), and Shakar‐95 (−31%). The complete phenotype dataset is attached as a supplemental file (Supplemental Table S2).

**TABLE 1:** Basic statistics and analysis of variance (ANOVA) for morpho‐physiological traits in control and cadmium (Cd) stress conditions

	Normal Conditions (T_1_)				Cadmium Stress (T_2_)					
Trait^a^	Min	Max	Mean	Gen^b^	Min	Max	Mean	Gen^c^	G × T^d^	Percent Reduction
PH (cm)	53.33	102.67	67.43	^***^	39.00	86.67	56.76	^***^	^***^	16.01%
Chl__till_	24.30	50.20	39.41	^**^	24.00	47.00	35.90	^**^	^***^	8.89%
Chl__head_	31.80	56.90	43.71	^**^	21.00	56.90	43.00	^**^	^***^	1.63%
BY (g/plant)	10.59	57.95	23.53	^**^	1.48	20.40	7.88	^**^	^***^	66.52%
TN	2.67	7.33	4.33	^**^	1.00	3.67	2.12	^**^	^***^	51.06%
SL (cm)	4.67	13.00	8.09	^**^	2.667	11.66	6.10	^**^	^***^	23.19%
TGW (g)	18.00	53.60	37.32	^***^	15.40	49.00	35.30	^***^	^***^	5.40%
GY (g/plot)	67.08	351.33	136.64	^***^	2.81	133.76	43.41	^**^	^***^	68.22%
GpS	8.00	61.00	30.65	^***^	6.00	91.00	28.23	^**^	^***^	7.89%
Fe (mg/kg)	10.10	39.63	21.88	^***^	0.28	7.42	1.41	^***^	^***^	93.54%
Zn (mg/kg)	3.97	59.00	30.00	^***^	0.02	3.44	2.13	^***^	^***^	92.90%
Leaf_Cd (mg/kg)	–	–	–	–	0.16	11.56	5.86	^***^	–	–
Seed_Cd (mg/kg)	–	–	–	–	0.16	29.53	14.85	^***^	–	–
STI	–	–	–	–	0.02	0.98	0.50	^***^	–	–

a Plant height (PH), Chlorophyll content at tillering (Chl__till_), Chlorophyll content at heading (Chl__head_), Biological yield per plant (BY), Tiller number (TN), Spike length (SL), Thousand grain weight (TGW), Grain yield per plot (GY), Grains per spike (GpS), Seed iron content (Fe), Seed zinc content (Zn), Leaf cadmium content (Leaf_Cd), Seed cadmium content (Seed_Cd), and Stress tolerance index (STI) in control and cadmium treated historical bread wheat panel

b Variance between genotypes within T_1_. Asterisks indicate statistical significance (^**^ = < .01, ^***^ = < .001)

c Variance between genotypes within T_2_. Asterisks indicate statistical significance (^**^ = < .01, ^***^ = < .001)

d Variance between genotypes in T_1_ and T_2_. Asterisks indicate statistical significance (^**^ = < .01, ^***^ = < .001)

**FIGURE 1:**
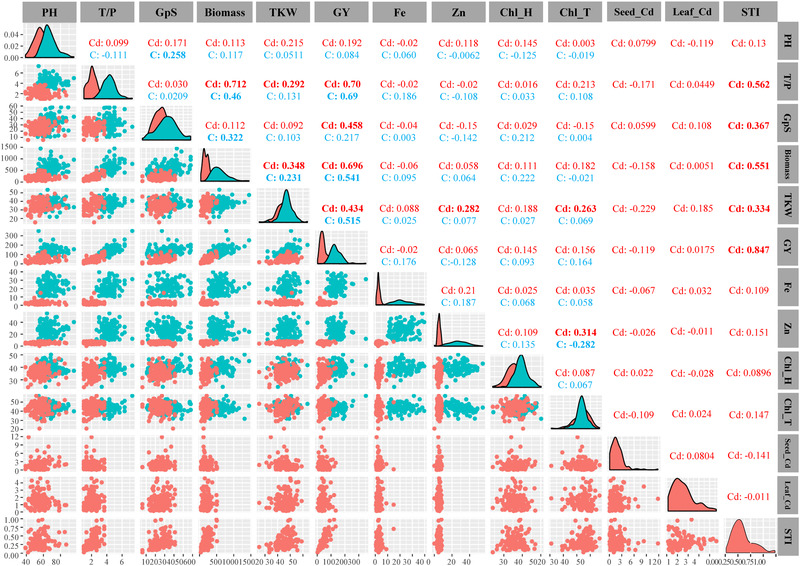
Scatterplots, histograms, and coefficient of correlation between traits under normal conditions (blue color) and Cd stress (red color). The lower triangle represents scatter plots of traits, the upper triangle represents coefficient of correlation values, and the middle line dissecting the two triangles represents histograms of traits. Trait nomenclature is presented in Table 1

Correlation analysis showed yield related traits in a strongly positive correlation with each other in both the treatments suggesting that associated traits were under the influence of same genetic factors. For instance, GY showed a positive correlation with BY (*r^2^* = 0.54), TGW (*r^2^* = 0.51) and TN (*r^2^* = 0.69) in T_1_. Similarly, in T_2_, GY showed a highly positive correlation with BY (*r^2^* = 0.69), GpS (*r^2^* = 0.45), TGW (*r^2^* = 0.43), and TN (*r^2^* = 0.71). STI also showed a positive correlation with all phenotypic traits in T_2_ including GY, TN, BY, GpS, and TGW with *r^2^* values of 0.84, 0.56, 0.55, 0.36, and 0.33, respectively (Figure 1).

### 3.2 Population structure, linkage disequilibrium, and association mapping

Population structure analysis using the rate of change of log probability between successive subgroup K values divided the panel into seven subgroups. Population structure estimation details were provided in our earlier reports on the diversity panel (Ain et al., 2015; Safdar et al., 2020), readers are encouraged to see those papers for further details. Population LD decay analysis showed highest LD in B sub genome (800 kb), followed by D sub genome (500 kb), and A sub genome (300 kb) (Supplemental Figure S1).

**FIGURE 2:**
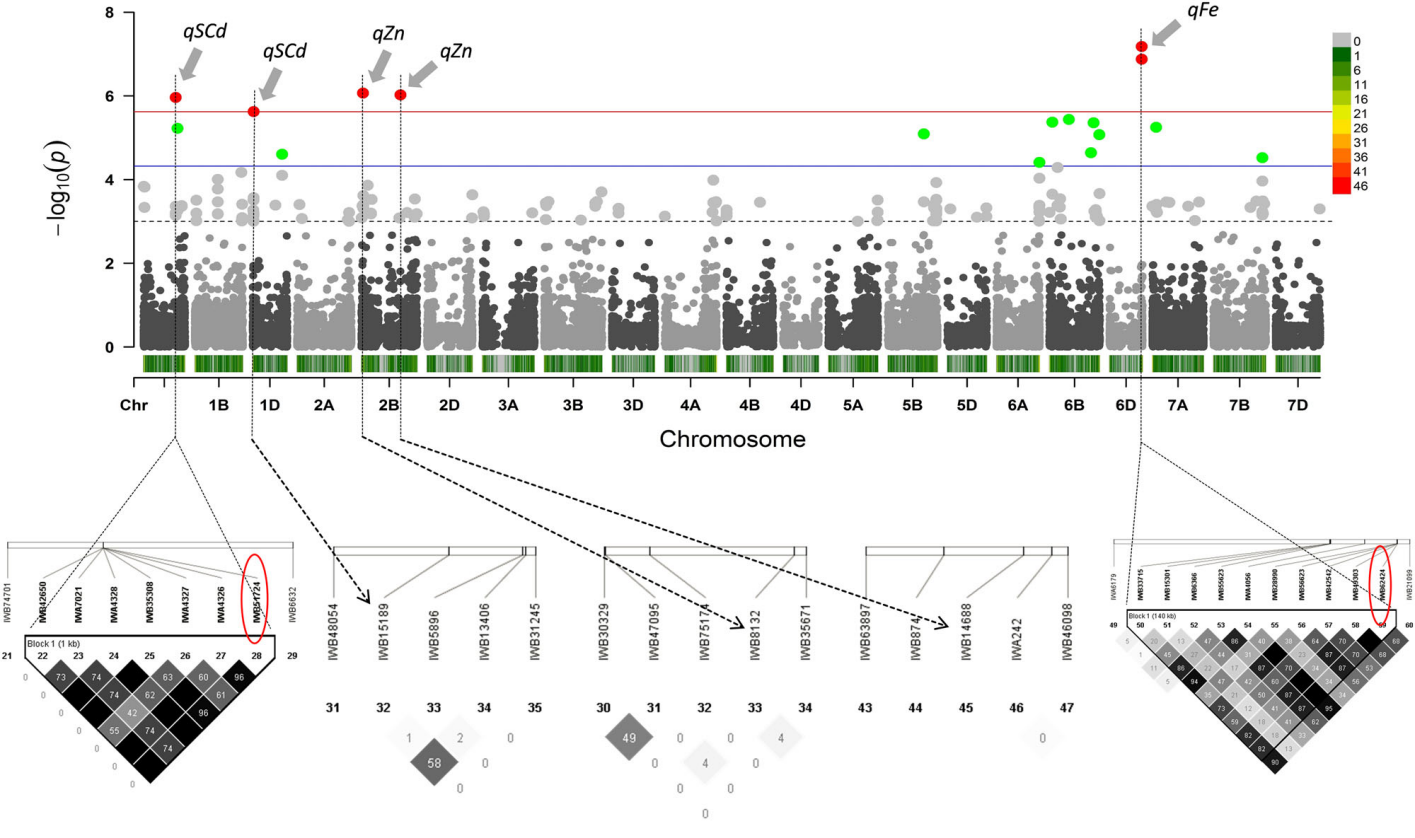
Manhattan plot of MTA identified by GWAS and LD blocks based on the pairwise LD between MTA and neighboring SNPs. The upper matrix represents Manhattan plot with three lines indicating three levels of statistical significance: dotted grey line (P < 0.001); blue line (P < 4.8E‐05); red line (P < 2.4E‐06). The lower matrix represents LD blocks of five statistically significant MTA based on pairwise LD with the neighboring SNPs. Trait nomenclature in Table 1

**FIGURE 3:**
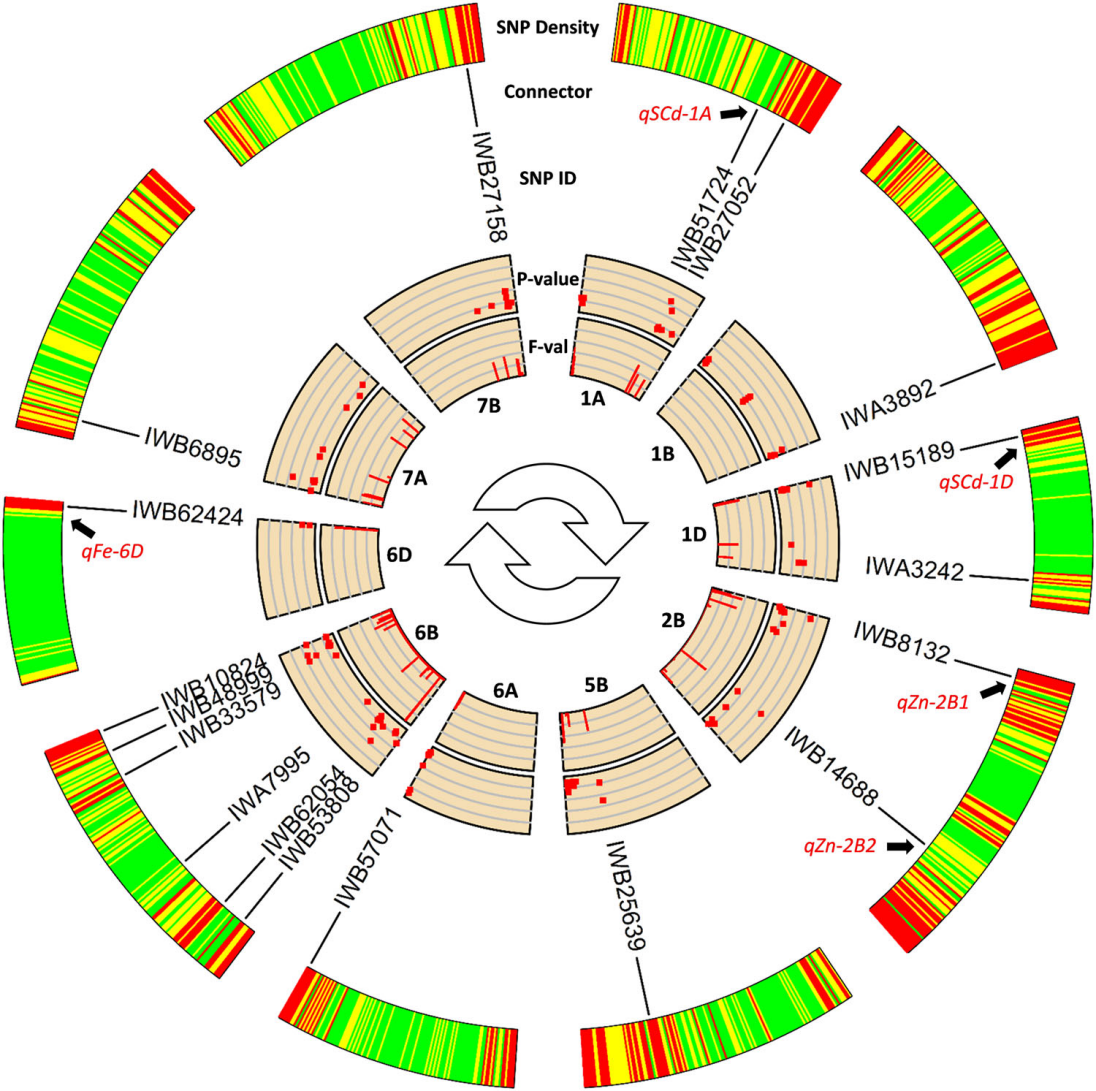
Genomic visualization of five loci and significant MTA identified at a significance threshold of 1/nSNP (‐log10p > 4.31). The outer circle represents SNP density from the 90K array data in the diversity panel. The middle circle represents all MTA identified on chromosomes harboring significant MTA; ‐log10p increases from inside out. The inner circle represents the additive effects of MTA; Add F value increases from inside out. Black arrows indicate representative SNPs of novel loci. The detailed description of MTA and loci is presented in Table S3 and Table 2, respectively

Genotype‐phenotype association by GWAS resulted in 179 marker‐trait associations (MTA) including 41 in T_1_, 81 in T_2_, and 57 in T_3_ at −log 10 *P *> 3 (Supplemental Table S3). The MTA detected under T_2_ were: Seed__Cd_ (14), BY (13), GY (12), PH (10), STI (9), GpS (5), TN (5), TGW (3), Fe (3), Chl__head_ (2), SL (2), Zn (2) and Leaf__Cd_ (1). Highest number of MTA were detected on B sub genome (46), followed by A sub genome (19), and D sub genome (16). MTA detected under T_1_ were: Chl__till_ (10), TN (10), GY (7), PH (7), TGW (4), and GpS (3) with highest number of MTA on A sub genome (25), followed by B sub genome (12), and D sub genome (2). Similarly, MTA detected under T_3_ were: TGW (18), GY (9), Zn (9), BY (8), SpS (5), TN (3), Fe (2), and PH (1), and Chl__till_ (1). Sub genomic distribution of MTA was: B sub genome (30), A sub genome (21), and D sub genome (6). Nineteen MTA were detected at a multiple testing correction of 1/nSNP (−log 10 *P* > 4.17) (Supplemental Table S4), while only five MTA (3 in T_2_ and 3 in T_3_) were detected at the genome‐wide significance of threshold 0.05/nSNP (−log 10 *P* > 5.47) (Figure 2). These five variants were identified for Seed__Cd_, Fe, and Zn on chromosome 1A, 1D, 2B, and 6D. All these five SNPs were present on different genomic regions with no linkage to each other. These loci are named according to nomenclature proposed by (McCouch, 1997) e.g., *qSCd‐1A* represents a genomic region at chromosome 1A for Seed__Cd_. It is located at 462435832 bp with −log 10 *P* = 5.96 and the tag SNP is IWB51724. The other four loci are *qSCd‐1D*, *qZn‐2B1*, *qZn‐2B2*, and *qFe‐6D* (Table 2). Genomic visualization of these statistically significant and novel loci is performed in RCircos package in R (Figure 3).

**TABLE 2:** Genomic regions identified by GWAS for the prediction of candidate genes

Locus	SNP^a^	Chr	Position^b^	Trait^c^	Treatment^d^	Add F	−log 10 P
*qSCd‐1A*	IWB51724	1A	462435832	Seed_Cd	T_2_	16.62	5.96
*qSCd‐1D*	IWB15189	1D	14985862	Seed_Cd	T_2_	15.35	5.62
*qZn‐2B1*	IWB8132	2B	25236559	Zn	T_3_	17.01	6.06
*qZn‐2B2*	IWB14688	2B	563182730	Zn	T_3_	17.07	6.02
*qFe‐6D*	IWB62424	6D	462605209	Fe	T_2_, T_3_	20.58	7.17

a SNP universal ID from IWGSC RefSeq v1.0.

b SNP physical Position from IWGSC RefSeq v1.0.

c Trait nomenclature is presented in Table 1 legends.

d Treatment details are presented in Supplemental Tables S2 and S3.

### 3.3 Gene prediction

Pairwise LD in the QTL regions (based on LD decay) of the five detected loci indicated that either the SNPs had no linkage with nearby SNPs or the LD block was too small (Figure 2). This could be due to the low marker density resulting from a small number of SNPs in the array dataset. This suggests that relying on pairwise LD to select the candidate region while dealing the array data may result in the loss of many potentially causal genes. Therefore, the genes were selected based on the sub genomic LD decay distance around the significant loci. Wheat reference assembly IWGSC RefSeq v.1.0 (Appels et al., 2018) database present at EnsemblPlant (http://plants.ensembl.org/Triticum_aestivum/Info/Index) was accessed to get the genes present in the five detected loci. This resulted in locating 208 genes (Table S5), among which 90 were regarded as IWGSC high confidence protein coding genes (Table S6). Limited information was found for these genes from the wheat genome annotation database, and hence the orthologous genes from rice and arabidopsis were extracted to get the annotation information. Based on the annotation information for the high confidence protein coding genes, many of the genes encoded proteins putative to the locus‐associated traits (Cd, Zn, and Fe). These included plant peroxidase, acetyltransferase, hexokinase, glycosyltransferase, and zinc‐finger proteins which have been associated with the genetic regulation of Cd in plants (Nazar et al., 2012; Whitt et al., 2018). Potentially important genes based on the information from annotations and literature are listed in Table 3.

**TABLE 3:** A list of potential genes associated with the uptake of Cd and other minerals in plants. These genes are shortlisted based on information from annotations and literature

Gene ID	At Ortholog^a^	Os Ortholog^b^	Annotation^c^
*TraesCS1A02G267200*	*AT1G33430*	*Os05g0427200*	Hexosyltransferase
*TraesCS1D02G034300*	*AT3G23560*	*Os07g0502200*	Protein DETOXIFICATION
*TraesCS1D02G034600*	*AT3G57380*	*Os01g0498300*	Glycosyltransferase
*TraesCS1D02G034900*	*AT3G09410*	*Os05g0111900*	Pectin acetylesterase
*TraesCS2B02G050900*	*AT5G43440*	*Os08g0391700*	1‐aminocyclopropane‐1‐CO homolog 9
*TraesCS2B02G051100*		*Os08g0392100*	2OG‐Fe(II) oxygenase domain containing protein
*TraesCS2B02G051700*		*Os04g0105100*	Zinc finger, RING/FYVE/PHD‐type domain
*TraesCS2B02G051800*	*AT2G28450*	*Os04g0105000*	Zinc finger CCCH domain‐containing protein 24
*TraesCS2B02G052300*	*AT1G67220*	*Os01g0246100*	Histone acetyltransferase HAC2
*TraesCS2B02G052600*	*AT5G40900*	*Os07g0294800*	Glycosyltransferase
*TraesCS2B02G397300*		*Os04g0519700*	Auxin response factor
*TraesCS6D02G383000*	*AT5G64790*	*Os06g0590600*	Beta‐1,3‐glucanase
*TraesCS6D02G383700*	*AT5G58940*	*Os02g0821400*	Calmodulin‐binding receptor‐like kinase 1
*TraesCS6D02G383800*	*AT4G00335*	*Os11g0294600*	Probable E3 ubiquitin‐protein ligase RHB1A
*TraesCS6D02G383900*		*Os10g0134900*	NB‐ARC domain containing protein
*TraesCS6D02G384100*		*Os10g0134900*	NB‐ARC domain containing protein
*TraesCS6D02G384300*	*AT4G00350*		Protein DETOXIFICATION
*TraesCS6D02G384400*	*AT4G00350*	*Os02g0821600*	Protein DETOXIFICATION

a Homologous gene in *Arabidopsis thaliana*.

b Homologous gene in *Oryza sativa*.

c Annotations are extracted from EnsemblPlant database for wheat, rice and arabidopsis.

### 3.4 Dissection of diversity panel based on Cd uptake and translocation

Cd uptake in leaf and translocation to seed varied significantly in the diversity panel, helping us dissect the panel into three subgroups: (1) hyper‐accumulators (Leaf__Cd_ high, Seed__Cd_ low) e.g. Bakhar‐02 (10.78 mg kg^−1^ Leaf__Cd_, 0.78 mg kg^−1^ Seed__Cd_), (2) hyper‐translocators (Seed__Cd_ high, Leaf__Cd_ low) e.g. Rohtas‐90 (29.53 mg kg^−1^ Seed__Cd_, 4.53 mg kg^−1^ Leaf__Cd_), and (3) moderate lines (low Seed__Cd_ and Leaf__Cd_) e.g. Lassani‐08 (0.15 mg kg^−1^ Seed__Cd_, 0.31 mg kg^−1^ Leaf__Cd_). Further details are provided in Table 4.

**TABLE 4:** Dissection of diversity panel based on Cd uptake and translocation

Group^a^	Genotype^b^	Seed__Cd_ (mg/kg)	Leaf__Cd_ (mg/kg)
Hyper‐accumulator	Bhakkar‐02	0.78	10.78
Hyper‐accumulator	BWP‐00	2.96	8.59
Hyper‐accumulator	Fareed‐06	4.53	8.75
Hyper‐accumulator	SH‐02	2.5	11.56
Hyper‐translocator	Anmol‐91	20.93	8.75
Hyper‐translocator	Fk Sarhad	17.5	1.87
Hyper‐translocator	Rohtas‐90	29.53	4.53
Hyper‐translocator	Zardana	15.78	4.37
Moderate Line	Lasani‐08	0.15	0.31
Moderate Line	Pasban‐90	0.46	0.15
Moderate Line	Rashkoh‐05	0.78	1.25
Moderate Line	Shakar‐95	0.78	0.15

a Groups are based on Cd uptake in leaf and seed; details are given in the results section.

b Genotypes belong to the Pakistani spring wheat diversity panel; details are given in Supplemental Table S1.

### 3.5 Effects of favorable alleles on leaf__Cd_ and seed__Cd_

**FIGURE 4:**
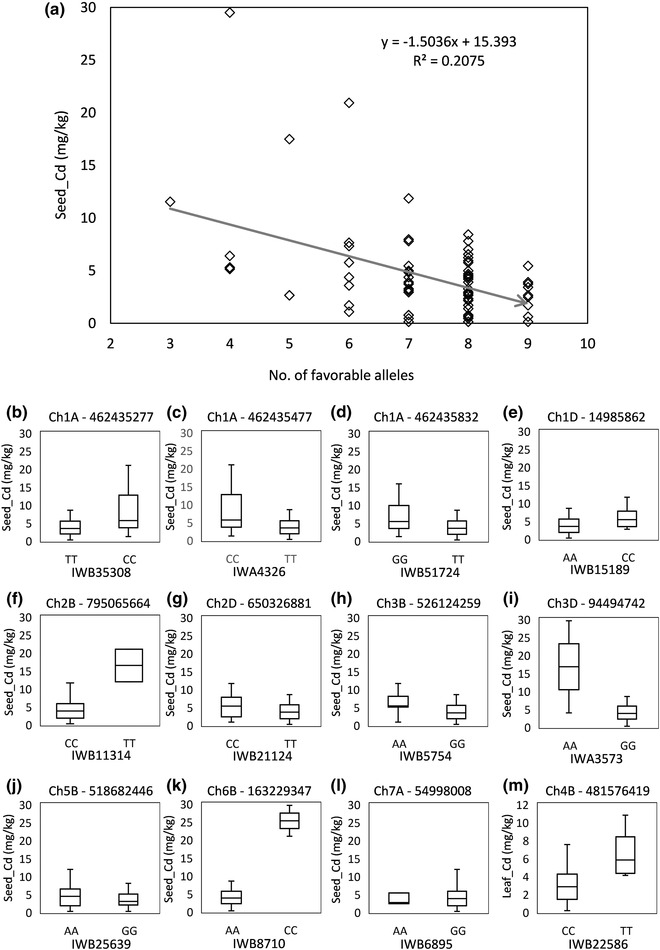
a) Linear regression between the number of favorable alleles and the seed cadmium concentration (mg/kg). b‐m) Box plots of MTA associated with cadmium accumulation in leaf and seed (mg/kg). Chromosome and position (bp) of SNPs are given on the top of each box‐and‐whisker. Boxes represent the first quartile, the median, and the third quartile, respectively. The thick horizontal lines correspond to the mean. Whiskers represent variability outside the upper and lower quartiles

The alleles of the significantly associated SNPs to Seed__Cd_ had a minor additive effect on the cadmium concentration in seeds. Seed__Cd_ concentration decreased with an increase in the number of favorable alleles. Linear regression (*r* ^2^) between the number of favorable alleles and phenotype was 0.20. There was no selection pressure on any of these favorable alleles as they were evenly distributed across old and modern cultivars (Figure 4a). The allelic distribution in SNPs associated to leaf and seed Cd showed that the favorable alleles had a significant impact on phenotype (Figure 4b‐m).

## 4 DISCUSSION

The present study describes the physiological implications of Cd toxicity in bread wheat and locates several potential loci for identifying Cd resistant genes. We observe that a high Cd uptake and accumulation in plants drastically lowers the uptake of other essential micronutrients like Fe (−93.5%) and Zn (−92.9%) attributing to the fact that Cd is transported by the same channels used for the Fe, Zn, and Mg uptake (Papoyan, Pineros, & Kochian, 2007; Roth, von Roepenack‐Lahaye, & Clemens, 2006). Therefore, identifying Cd resistant genes is not only important to ensure edible quality of the grain but is also critical to maintain the mineral composition of the plant. In our diversity panel, the majority of genotypes expressed more than an 80% reduction in Fe uptake and a more than 75% reduction in Zn uptake, under Cd stress. Such deficiency of these micronutrients, especially Fe, causes severely damaging effects on different biological and physiological processes in plants including photosynthesis. Photosynthetic rate of a plant significantly decreases with the damage of photosystem II (PSII) under heavy metal stress, which results in an impaired plant growth (Rodriguez‐Serrano et al., 2009; Tanyolac, Ekmekci, & Unalan, 2007). This damage of PSII may also involve a significant reduction in the chlorophyll content—an 8% reduction in the chlorophyll content is observed here at the tillering stage. Likewise, Cd toxicity also has unfavorable effects on other agronomic traits. For example, a reduction of 66% in BY is observed under Cd stress in this study, which is similar to an earlier report ofDias et al. (2013), where higher Cd concentrations are noticed to reduce the overall plant biomass by 76%. Such large‐scale reductions in the crop productivity by Cd toxicity can potentially challenge the continuously increasing demand of food crops needed to feed an exponentially growing global population. Cd toxicity is so critical that it damages the plant chemistry even when the plant otherwise looks normal. For example, two varieties in our panel (SKD‐1 and TD‐1) retained a normal growth in PH, TN, and GY under Cd stress and were considered resistant. Although these two varieties maintained normal growth, the grain Fe and Zn concentrations were both reduced by more than 80% in these varieties. All these findings indicate that Cd not only hampers grain yield or toxifies the grain, but also demineralizes the grains and reduces their overall nutritional quality. However, these resistant varieties can be used in conventional breeding approaches along with low‐Cd and optimum mineral uptake varieties to breed nutritional and healthy wheat cultivars.

GWAS analysis performed here identified several potential SNP‐trait associations for Cd uptake and other agronomic traits under Cd stress. The MTA associated with agronomic traits as well as leaf and seed Cd were randomly distributed among landraces, green revolution, post green revolution, and modern cultivars, suggesting that they have not been targeted in any breeding programs. The cultivars having a higher number of favorable alleles can be used as a breeding resource and the present results may provide a basis to initiate genomic selection methods to maximize genetic gains (Jannink, Lorenz, & Iwata, 2010). Therefore, they should be combined in future breeding programs for low or no Cd, just like *{Rasheed, 2016 #3}Ppd‐D1, Rht‐B1, Rht‐D1, TaGW2‐6A*, and *TaCKX6‐D1* in Pakistani wheat cultivars (Rasheed et al., 2016). The excluder mechanism rendering low or zero Cd in grains is of significant importance and must be fully explored in future studies.

Most of the MTA identified here are mapped under the previously reported QTL either in wheat or its grass relatives. Among the ten MTA identified for PH in T_2_, four present on 2B, 4B, 5B, and 6B are positioned under a previously identified QTL for shoot height on the orthologous chromosomal positions (Xue, Chen, & Zhang, 2009). The *Rht1* locus on 4B, which was a major breakthrough against lodging as part of the green revolution, is identified in both experimental conditions in the present study. MTA for BY identified on chromosome 1B is present under a QTL for shoot fresh weight under Cd stress in wheat (Ci et al., 2012). Similarly, MTA for Cd concentration on chromosome 1A, 1D, 2B, 2D, 3B, 3D, and 6B are present under a QTL for Cd uptake on the orthologous position in rice (Wang et al., 2018; Xue et al., 2009). Some loci identified in present study are located in the regions of already cloned Cd resistant genes in rice. For example, MTA for STI, GY, BY, and Seed__Cd_ on chromosomes 1A, 1B, and 1D are located in the region of *OsMTP1* (Yuan, Yang, Liu, Zhang, & Wu, 2012) and MTA for Seed__Cd_ and TGW detected on chromosomes 2A and 2D are present in the orthologous position of *OsPCR1* (Song et al., 2015). Five MTA on 5B (IWB29686, IWB39994, IWB2735, IWB12428, and IWB66179) are identified in the region of *Cdu‐B1* (5B: 695659830–696420845) which is reported as a major QTL controlling Cd uptake in wheat (Knot et al. 2009). These five MTA are associated to GY under T_2_ (Cd stress) and their −log10 *P* values range from 3.19 to 3.92 (Table S3), none of which could pass the stringent criteria of multiple testing corrections along with many other important MTA discussed above. Although the five MTA present in the region of *Cdu‐B1* demonstrate the role of this important locus in the uptake of Cd in the diversity panel, it is unclear if the same QTL and candidate gene are causing the associations in this study. These findings suggest that the stringent criteria of Bonferroni correction, used so frequently in GWAS studies, result in the loss of many potential genetic variants (Gordon et al., 2016). However, this is not the first time that *Cdu‐B1* has been found as a minor effect locus in a GWAS study for Cd uptake in bread wheat. Another possible reason, apart from the statistical stringency, could be the polygenic and complex nature of this trait and the genotypes of different genetic background used, as has been suggested in earlier studies where *Cdu‐B1* was found as a minor effect locus (Bhatta et al., 2018). Another important SNP on 5B (IWB25639) was identified as associated with Seed__Cd_ with a −log10 *P* value of 5.092. This SNP had two alleles as AA and GG in the diversity panel. Genotypes having allele GG show a lesser uptake of Cd as compared to the genotypes with allele AA. Recently, Maccaferri et al. (2019) have reported a Cd/Zn transporter (*HMA3*) on chromosome 5B with two alleles; *TdHMA3‐B1a* and *TdHMA3‐B1b*. First transports Cd to the vacuoles in the roots thus limiting its uptake to shoot, while the later increases Cd uptake to the aerial plant parts in durum wheat. IWB25639 on 5B showing significant genotypic variation in Cd uptake for its two alleles may have been capturing this *HMA3* transporter; however, this too could not fulfill the significance criterion of Bonferroni. This again suggests that the statistically stringency of multiple testing correction to remove false positives emerging from type‐I error may result in false negatives caused by type‐II error. Therefore, other factors such as consistency over experimental environments or association models and the relevance of underlying genes with the target traits should also be considered before ruling out the significantly associated SNP.

Five loci that passed the multiple testing correction do not colocalize with any of the reported QTL for cadmium uptake in wheat, and hence should be further validated and investigated for candidate genes using other functional genomics approaches. Genes present in these loci encode proteins including glucosyltransferase, acetyltransferase, zinc‐finger, and others that have been associated with minerals (particularly Cd) uptake and regulation (Nazar et al., 2012; Whitt et al., 2018). An important locus on chromosome 2B (*qZn‐2B1* – IWB8132) has six genes that encode *2OG‐Fe*, *zinc finger CCCH‐type*, *zinc finger RING/FYVE/PHD‐type*, and *glucosyltransferase* proteins suggesting that it could potentially be involved in the uptake of Cd since Cd uptake takes the same route as that of Fe and Zn (Papoyan et al., 2007; Roth et al., 2006). Further research would require a careful and deep understanding of the evolutionary history of these genes and their physiological role in the relative grass species, considering the complexity and polygenic nature of Cd uptake in plants.

## 5 CONCLUSIONS

The present study provides a comprehensive information on genetic variations associated with Cd toxicity and uptake in a historical bread wheat diversity panel from Pakistan. Five novel loci associated with Cd, Fe, and Zn uptake are identified and their associated regulatory genes are studied in reference to their established mechanisms studied or reported in other crops. Candidate genes underlying these loci are potential targets for functional genomics approaches to develop high yielding and contamination free wheat varieties. Further, morphophysiological screening of the diversity panel has identified Cd stress resistant lines (SKD‐1 and TD‐1), which sustain their grain yield under stress although accumulating high Cd concentration in seeds. These lines can be used with moderate lines (low Cd uptake lines) in conventional breeding programs to produce resistant and healthy wheat cultivars. Thus, this study not only provides an invaluable information for functional genomics through candidate gene mining, it also supplies material for varietal development in stress affected areas.

## DATA AVAILABILITY

The phenotype dataset used in this study is available in the supplemental file (Supplemental Table S2). The genetic variation dataset can be found in the European Variation Archives (EVA), Project: PRJEB36127; Analyses: ERZ1283759.

## AUTHORS CONTRIBUTION STATEMENT

UMQ designed the study. LBS, FA, SS and ME participated in phenotyping. LBS, FA, SS and ZA participated in the laboratory experimental work. LBS and FA performed the statistical analysis and visualizations. LY, MMT and MI provided assistance in data analysis. LBS drafted the initial manuscript. UMQ, SL and ZM edited the manuscript. UMQ and SL provided the necessary resources and supervised the study.

## CONFLICT OF INTEREST

The authors declare no conflict of interest.
